# Nasal molding prevents relapse of nasal deformity after primary rhinoplasty in patients with unilateral complete cleft lip: An outcomes‐based comparative study of palatal plate alone versus nasoalveolar molding

**DOI:** 10.1002/cre2.502

**Published:** 2021-10-24

**Authors:** Yukiko Aihara, Toru Yanagawa, Masahiro Sasaki, Kaoru Sasaki, Yoichiro Shibuya, Koji Adachi, Shinji Togashi, Shohei Takaoka, Katsuhiko Tabuchi, Hiroki Bukawa, Mitsuru Sekido

**Affiliations:** ^1^ Department of Plastic and Reconstructive Surgery Faculty of Medicine, University of Tsukuba Ibaraki Japan; ^2^ Department of Oral and Maxillofacial Surgery Faculty of Medicine, University of Tsukuba Ibaraki Japan; ^3^ Department of Molecular and Cellular Physiology Institute of Medicine, Academic Assembly, Shinshu University Nagano Japan

**Keywords:** cleft lip and palate, Hausdorff distance, presurgical nasoalveolar molding, presurgical orthopedic treatment

## Abstract

**Objectives:**

In recent years, many studies have reported that the presurgical nasoalveolar molding method improves the nose morphology; however, the reason for its effectiveness after surgery has never been understood. We evaluated the effect of nasoalveolar molding by comparing it with a passive orthopedic method without a nasal stent and focusing on the nostril morphology after primary cheiloplasty using various measurement methods. We then analyzed the essential factors.

**Materials and methods:**

The patients involved were 31 infants with unilateral complete cleft lip and palate treated with primary cheiloplasty at the University of Tsukuba Hospital from 2004 to 2011. Of the 31 infants, 16 received nasoalveolar molding treatment and 15 received passive orthopedic treatment as controls. Photographic facial measurements were performed for all patients immediately and 7 months after primary cheiloplasty. The esthetics of the nostrils were assessed according to the left–right nostril symmetry, as measured by the Hausdorff distance, area ratio, perimeter ratio, and aspect a/u (the aspect ratio of the affected side)/(the aspect ratio of the unaffected side) ratio. In addition, the inclination of the nasal ridge was assessed using anthropometric measurements (Grc‐Grn∠midline and midline∠columellar axis).

**Results:**

The area ratio, perimeter ratio, and Grc‐Grn∠midline were significantly greater in the nasoalveolar molding group immediately after surgery (*p* = 0.00062, 0.016, and 0.048, respectively) than in the control group. However, the Hausdorff distance and aspect a/u ratio were more favorable (*p* = 0.0018 and 0.0039, respectively) in the nasoalveolar molding group after 7 months.

**Conclusions:**

The results of our study suggested that using nasoalveolar molding as a presurgical orthopedic treatment could improve the shape of the nasal cartilage with surgeon's corrections.

## 1 INTRODUCTION

In recent years, the usefulness of presurgical orthopedic techniques has been analyzed from various perspectives. It has been reported that such techniques could improve the management and short‐term outcomes of cleft lip and palate (CLP) surgery, prevent developmental delays in infancy, and help normalize deglutitive functions (Gnoinski, 1990). These techniques also facilitate the surgical improvement of the nasal morphology by minimizing distortion of the dental arch and tension at the surgical site (Gnoinski, 1990). Long‐term improvements in speech, maxillofacial growth, and esthetics have also been reported (Prahl et al., 2001).

The nasoalveolar molding (NAM) method has been studied as a conventional presurgical orthopedic treatment. NAM has been increasingly used in the treatment of CLP to improve surgical, esthetic, functional, and socio‐economic outcomes before undertaking primary cheiloplasty (Maillard et al., 2017). In the presence of unilateral CLP, there is some evidence that NAM may improve the surgical outcome of the nasal morphology, although comparative data with other techniques are limited. Despite the relative paucity of high‐level evidence, NAM could be a promising technique (Abbott & Meara, 2012). Especially, there is a trend of improved symmetry of the nostril shape, although this may not always be the case (van der Heijden et al., 2013). In patients with CLP, deformity of the nose after primary cheiloplasty is a significant esthetic issue (Mommaerts & Nagy, 2008). Various methods are used to assess the shape of the nose and its functions in patients with CLP, such as two‐dimensional photographs to determine specific points using anthropometric measurements, use of models, and use of three‐dimensional technologies (Hosseini et al., 2017; Maillard et al., 2017). In previous studies, NAM stents were removed at a fixed time; however, the effect of NAM persisted even after the nasal cartilage was no longer supported by the stents, and the relapse rate was also less (Barillas et al., 2009). The nasal stents were found to improve clinical outcomes before (Isogawa et al., 2010; Kozelj, 1999; Monasterio et al., 2013; Punga & Sharma, 2013) and after surgery (Lopez‐Palacio et al., 2012; Nakamura et al., 2009; Sasaki et al., 2012). However, the essential factor in the NAM method that improves the shape of the nasal morphology remains unclear.

Previous reports have not described the specific surgical techniques used. Surgeons performed primary cheiloplasty to obtain more symmetrical outcomes of the nasal ala by using certain techniques, such as over‐ or under‐correction based on their own clinical experience. However, these studies had merely compared the shape of the nose at specific time points. Therefore, it is necessary to evaluate the effect of the NAM method immediately after surgery and after a certain period to clarify what the most important contributing factors are to successful CLP surgery. In this study, we used the Hausdorff distance, aspect ratio, perimeter diameter, and area ratio to evaluate the morphology of the nostrils and anthropometric measurements (Grc‐Grn∠midline and midline∠columellar axis) to evaluate the direction of nasal growth. Further, we analyzed the initial corrections made by the surgeons and clarified the factors determining the success of the NAM method.

## 2 MATERIALS AND METHODS

### 2.1 Ethics

All parents of the study participants provided informed consent, and the study design was approved by the appropriate ethics review board (approval no. H23‐87).

### 2.2 Patients and treatment

For this retrospective cohort study, 31 consecutive Japanese infants (19 males and 12 females) with unilateral CLP who underwent unilateral cheiloplasty at the University of Tsukuba Hospital between 2004 and 2011 were enrolled. None of the patients had other craniofacial malformations or systemic diseases. Of the 31 infants, 16 infants received presurgical orthopedic treatment using the presurgical NAM method (the NAM group) and 15 received passive orthopedic treatment with a modified Hotz plate (mHP, the mHP group) as controls. The NAM group included two patients with incomplete cleft lip, and the mHP group included one patient with Simonart's band. We have used the Hotz plate in our practice since 2004 for patients whose parents requested it, and NAM has been used since 2007. This study compared the outcomes after primary cheiloplasty between these two presurgical methods. The characteristics of the patients are summarized in Table 1.

**Table 1:** Characteristics of unilateral cleft lip and palate patients according to orthopedic treatment

	Modified Hotz plate group	Nasoalveolar molding group	*p* Value
Gender			
Male	6	13	
Female	9	3	
Male:Female ratio	1:1.5	1:4.3	0.023*
Affected side			
Left	10	7	
Right	5	9	
Left:Right ratio	1:0.5	1:1.3	0.35**
Mean age (days) of the staring appliance	11.1 ± 12.3	21.1 ± 22.6	0.15***
Mean age (days) at the time of primary cheiloplasty	116.5 ± 23.0	105.4 ± 20.5	0.16***
Mean period (days) of observation	228.4 ± 51.8	201.4 ± 37.0	0.10***
Mean treatment period (days) with orthopedic appliance before primary cheiloplasty	105.4 ± 27.6	85.6 ± 19.9	0.084***
Mean age (days) at the time of evaluation	345.0 ± 47.5	324.2 ± 37.5	0.18***
Mean treatment period (days) with orthopedic appliance	333.9 ± 47.6	305.9 ± 32.0	0.084***

*Note*: *Fisher's exact test; **chi‐square test; ***Student's *t*‐test.

### 2.3 Presurgical orthopedic treatment

**Figure 1:**
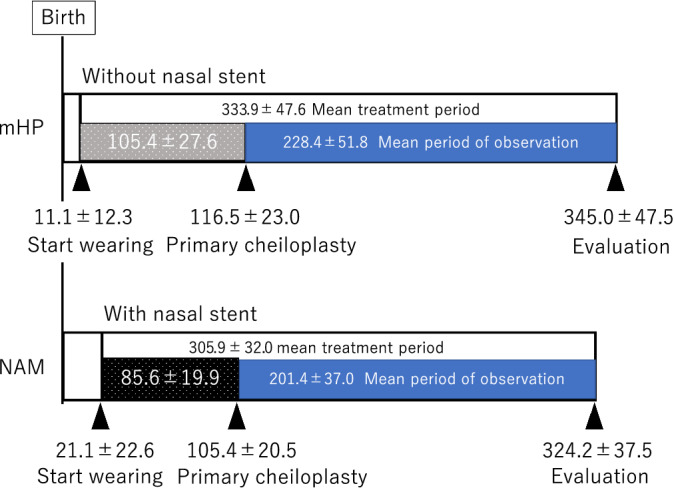
Scheme of the orthopedic treatment

The scheme of the orthopedic treatment is shown in Figure 1. The presurgical orthopedic treatment using presurgical NAM or the mHP was performed at the Department of Oral and Maxillofacial Surgery, Tsukuba University. An intraoral maxillary impression was obtained and applied to patients at the age of 11.1 ± 12.3 days and 21.1 ± 22.6 days in the mHP and NAM groups, respectively. The presurgical orthopedic procedures were performed as previously described (Adachi et al., 2013; Sasaki et al., 2012). The mHP was fabricated from soft and hard acrylic using a maxillary cast model, which was obtained at the patients' first visit. In the mHP procedure, resin processes in the spaces between the soft palate were scraped off and the same shape was fabricated with the NAM plate. The plate was worn constantly in combination with extraoral strapping of the upper lips until primary cheiloplasty and without strapping until palatoplasty. In the NAM procedure, the nasal stents were adjusted weekly to provide a gentle molding force to the lower lateral cartilage and nasal tip. A horizontal rubber band applied a force at the junction of the forming columella and prolabium in the posterior direction. The rubber band was always used during the period when the nasal stent of the NAM appliance was in use. The stents of the NAM appliance were positioned at the start of treatment and removed after the primary cheiloplasty. Similar palatal plates were used in both groups until the subsequent palatoplasty.

The mean treatment period with the orthopedic appliance in the NAM group was 303.7 ± 32.1 days, and that in the mHP group was 333 ± 47.6 days (Table 1). The orthopedic force was applied by the nasal stent with the rubber band for 85.6 ± 19.9 days in the NAM group.

### 2.4 Primary cheiloplasty and rhinoplasty

**Figure 2:**
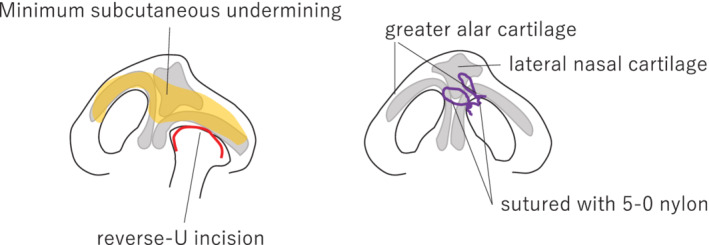
Diagrammatic representation of the rhinoplasty procedure

The mean age for primary cheiloplasty in the NAM group was 105.4 ± 20.5 days, and that in the mHP group was 116.5 ± 23.0 days (Table 1). All patients underwent cheiloplasty by a rotation advancement plus small triangular flap method (Onizuka procedure) with anatomical reconstruction of the orbicular oris muscle, as described previously (Adachi et al., 2013). All muscles and the nasal cartilage were corrected after suturing all muscles and flaps. Minimum subcutaneous undermining was performed over both alar cartilages using a reverse‐U incision on the affected side. After that, the right and left greater alar cartilages were sutured with 5‐0 nylon under non‐direct vision using a 23G needle. In cases where there was a large deviation, a lifting suture was added to the lateral nasal cartilage on the unaffected side (Figure 2). A silicon nostril retainer (Koken Co., Tokyo, Japan) was used for more than a month. The same surgeon (S.T.) performed all surgeries.

### 2.5 Evaluation of the surgical outcome of the nostril morphology

#### 2.5.1 Photography and measurements

All photographs were taken using a standardized handheld technique with the same digital camera from the midline of the face perpendicular to the face and from below the face at an elevation angle of 30° to the face (by Y.A., S.T., and M.S.). A series of frontal‐view photographs were taken for each patient during primary cheiloplasty and after approximately 7 months (mHP, 228.4 ± 51.8 days; NAM, 201.4 ± 37.0 days) after primary cheiloplasty. Based on these photographs, we standardized the object and performed subsequent measurements and analyses (Adachi et al., 2013). Two people (T.Y. and K.T.) determined the anthropometric landmark points and extraction of curve of the nostril contour in the photographs.

#### 2.5.2 Measurement of the Hausdorff distance

The Hausdorff distance was calculated as described previously (Karube et al., 2012). This is a virtual distance that measures the distance between two subsets of a metric space. The score is 0 if two objects are identical while the score is more if the shapes of the objects are different. In brief, we plotted the outline of each nostril using Canvas 11 J with geographic information system (GIS) (ACD Systems International Inc., Canada). The shape of the nostril on both sides was extracted and converted to a GIS. It was analyzed with PostGIS—an open‐source software program that adds support for geographic objects to the PostgreSQL object‐relational database—and the Hausdorff distance of the shape of the nostril on both sides was measured.

If *X* and *Y* are two non‐empty subsets of a metric space (*M*, *d*), the Hausdorff distance *d*_H_ (*X*, *Y*) is:

$$d_{H}\left(X,Y\right)=\max\left\{\underset{x\in X}{\sup}\underset{\;y\in Y}{\inf}d\left(x,y\right),\underset{y\in Y}{\sup}\underset{\;x\in X}{\inf}d\left(x,y\right)\right\}$$

where, *sup* represents the supremum and *inf* the infimum.

Equivalently,

$$d_{H}\left(X,Y\right)=\inf\left\{\in >0;X\subseteq Y_{\in }\;\text{and}\;Y\subseteq X_{\in }\right\}$$

where

$$X_{\epsilon }:==\underset{x\in X}{U}\left\{z\in M;d\left(z,x\right)\leq \epsilon \right\}$$

is the set of all points within ε of the set *X* (sometimes called the epsilon‐neighborhood of X).

#### 2.5.3 Area ratio, perimeter ratio, and aspect a/u ratio

We extracted the shape of the nostril from photographs, measured the area and perimeter of the nostril using Image J version 1.42 (NIH, Bethesda) and then calculated the ratio between the affected and unaffected sides to determine the area and perimeter ratios, respectively. For the aspect ratio, we used Feret's diameter to measure the maximum axis as the long axis and the minimum axis as the short axis and calculated the (long axis)/(short axis) ratio. The aspect a/u ratio was determined as (the aspect ratio of the affected side)/(the aspect ratio of the unaffected side).

#### 2.5.4 Grc‐Grn∠midline and midline∠columellar axis

The anthropometric landmarks were determined, as described by Nagy and Mommaerts (2007). The most superior point of the nasal alar groove (Gr) was added as described in a previous report (Adachi et al., 2013). Briefly, the facial midline was determined as the line perpendicular to the line connecting both endocanthion points that bisected it at the midpoint. The angle between the line connecting the Gr point at the cleft side (Grc) and the Gr point at the non‐cleft side to the facial midline was defined as the Grc‐Grn line∠midline. The pronasal (Pn) is the most superior point on the outline of the tip of the nose, and the columellar axis was defined as the line connecting the Pn and midpoint of the line connecting the most medial points of the inner border of the nostril. The midline∠columellar axis was defined as the angle between the facial midline and columellar axis. The anthropometric landmarks and constructs were plotted on the photographs and analyzed using Image J (version 1.42) software (National Institutes of Mental Health, Maryland, USA).

### 2.6 Statistical analyses

The Fisher's exact, *χ*‐square, and Mann–Whitney *U* tests were used to analyze gender, the left and right sides of the disease site, and the aspect a/u ratio, respectively. Since the remaining factors examined in this study—the Hausdorff distance, area ratio, perimeter ratio, Grc‐Grn∠midline, and midline∠columellar axis scores—were considered to follow a normal distribution, a Student's t‐test was performed for the analysis between the NAM and mHP groups. Statcel 4.0 (OMS Ltd., Tokyo, Japan) was used for statistical analyses. P values less than 0.05 were considered statistically significant.

## 3 RESULTS

### 3.1 Photographs of typical cases

**Figure 3:**
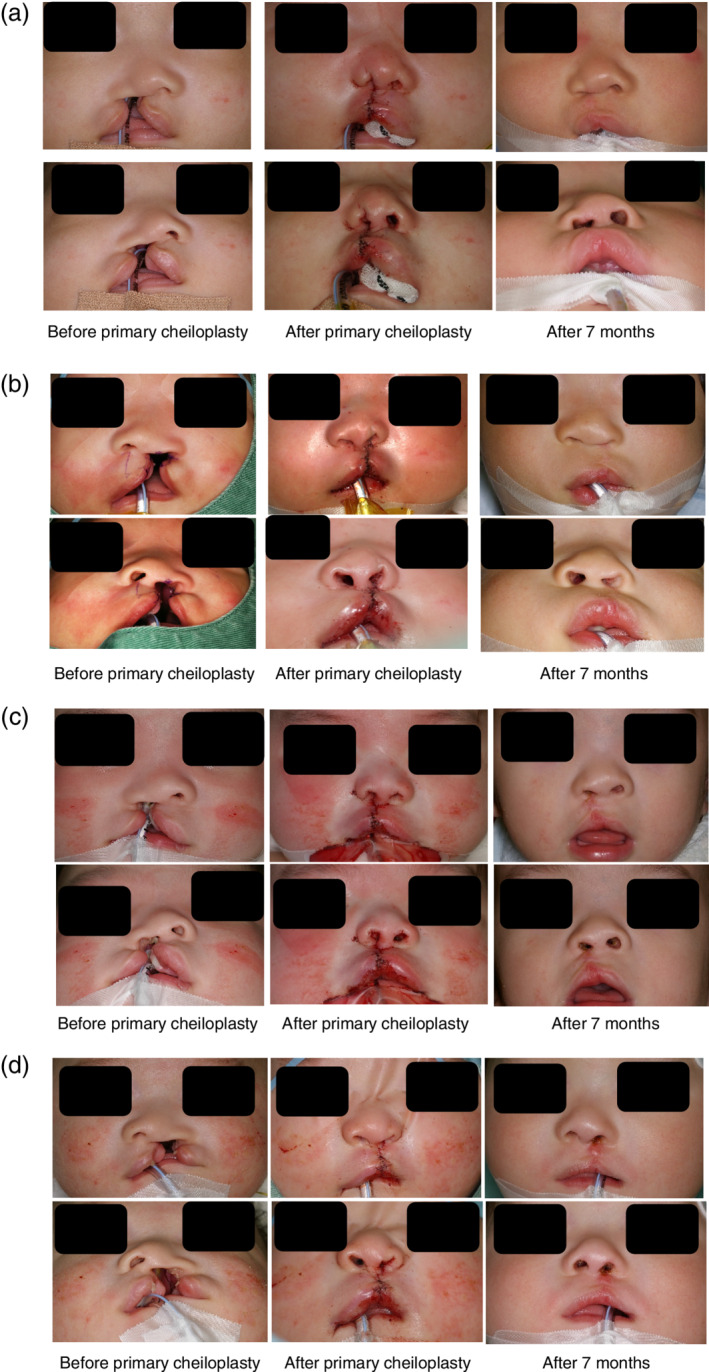
Photographs of typical cases where the patients were in either the Hotz plate (mHP) or nasoalveolar molding (NAM) group. Photographs were taken from the midline of the face perpendicular to the face (top row), and from below the face at an elevation angle of 30° to the face (bottom row). (a, b) Photographs of mHP patients' noses before, after, and 7 months after primary cheiloplasty. (c, d) Photographs of NAM patients' noses before, after, and 7 months after primary cheiloplasty

Photographs of the nostril morphology of before primary cheiloplasty, after primary cheiloplasty, and after 7 months were shown (Figure 3a–d).

### 3.2 Comparison of the nostril morphology immediately and 7 months after surgery

#### 3.2.1 Hausdorff distance

**Figure 4:**
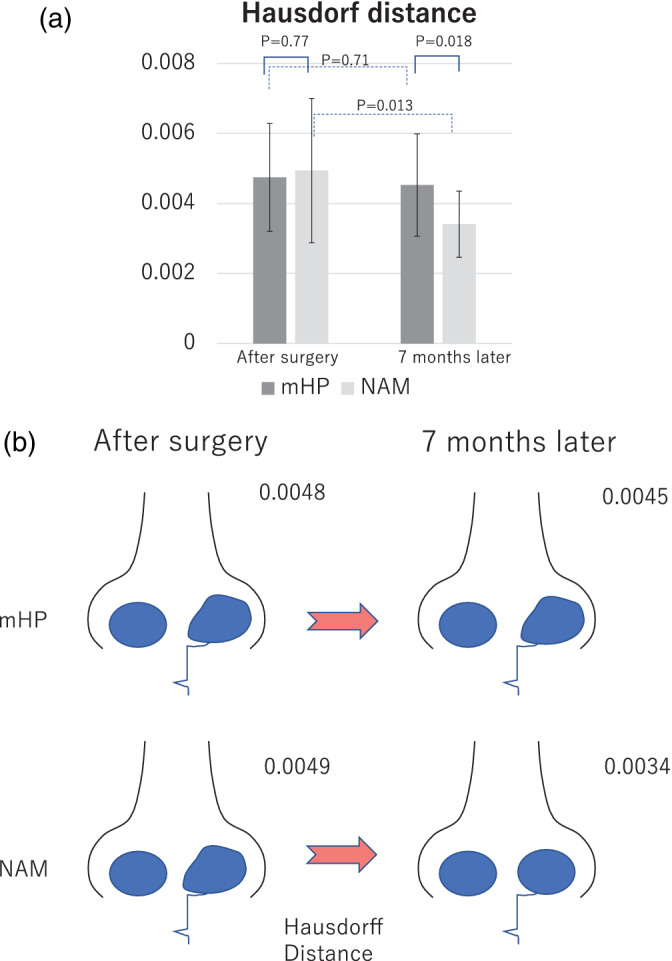
Evaluation of the surgical outcome in the modified Hotz plate (mHP) and nasoalveolar molding (NAM) groups analyzed by the Hausdorff distance. (a) Temporal change in the surgical outcome in the two groups. mHP: mHP group (dark gray), treatment with mHP. NAM: NAM group (light gray), treatment with NAM. Results immediately and 7 months after surgery are shown. (b) Conceptual schema of the time‐series changes in the nasal morphology and relationships with the NAM and mHP groups

The Hausdorff distance is a virtual number indicating the similarity of two sets. The Hausdorff distance value in the mHP group decreased from 0.0048 to 0.0045, while that in the NAM group decreased from 0.0049 to 0.0034. The Hausdorff distance of the NAM group decreased significantly (*p* = 0.013), whereas that of the mHP group did not change (*p* = 0.71), indicating that the affected nostril of the NAM group was similar to that of the unaffected group (Figure 4a,b).

#### 3.2.2 Area ratio

**Figure 5:**
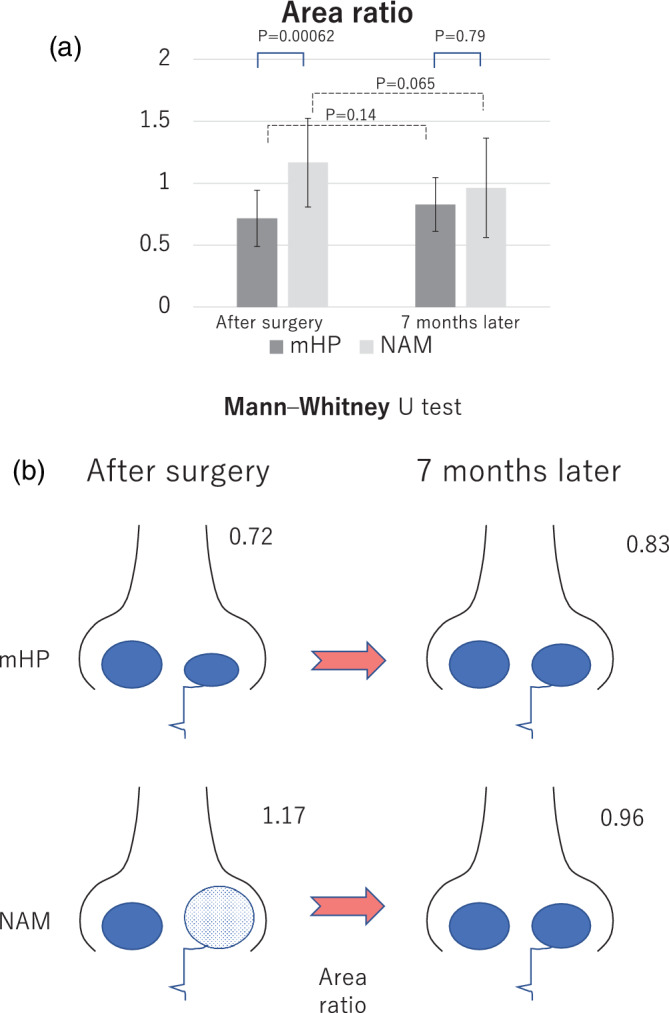
Evaluation of the surgical outcome in the modified Hotz plate (mHP) and nasoalveolar molding (NAM) groups analyzed by the area ratio. (a) Temporal change in the surgical outcome in the two groups. mHP: mHP group (dark gray), treatment with mHP. NAM: NAM group (light gray), treatment with NAM. Results immediately and 7 months after surgery are shown. (b) Conceptual schema of the time‐series changes in the nasal morphology and relationships with the NAM and mHP groups

The area ratio is the ratio of the area of the normal side to that of the affected side. The closer the value is to 1, the more identical it is. Hence, the ideal ratio is 1.0, which indicates a similar area of the affected and unaffected sides. The area ratio of the mHP group increased from 0.72 to 0.83, while that of the NAM group decreased from 1.17 to 0.96; thus, it became close to 1.0 in both groups. The area of the mHP group was increased, while that of the NAM group was decreased; however, there was no significant difference between the results immediately and 7 months after surgery (*p* = 0.14, *p* = 0.065, respectively; Figure 5a,b).

#### 3.2.3 Perimeter ratio

**Figure 6:**
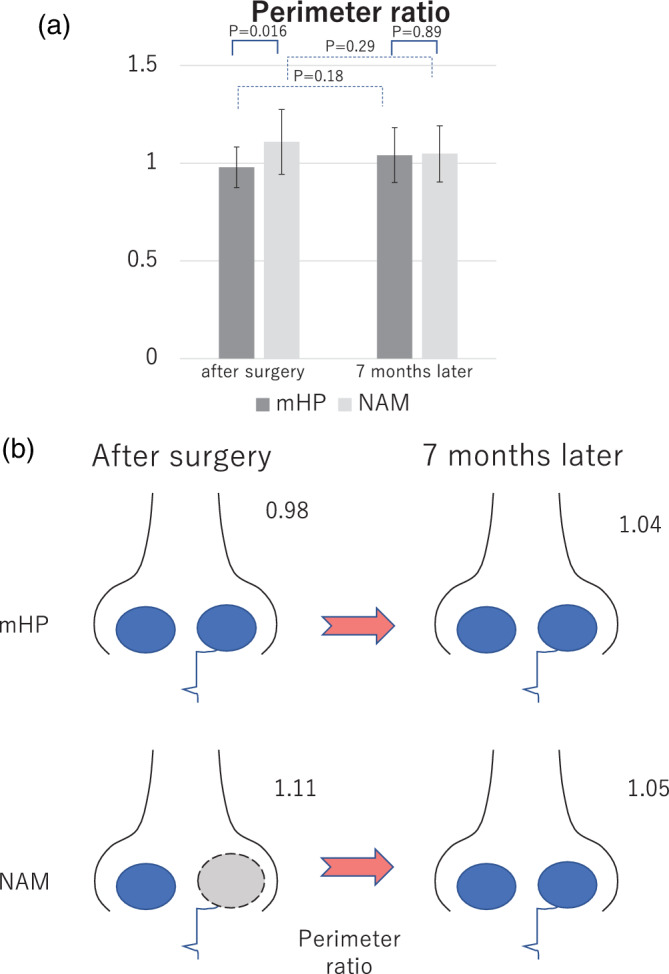
Evaluation of the surgical outcome in the modified Hotz plate (mHP) and nasoalveolar molding (NAM) groups analyzed by the perimeter ratio. (a) Temporal change in the surgical outcome in the two groups. mHP: mHP group (dark gray), treatment with mHP. NAM: NAM group (light gray), treatment with NAM. Results immediately and 7 months after surgery are shown. (b) Conceptual schema of the time‐series changes in the nasal morphology and relationships with the NAM and mHP groups

The perimeter ratio signifies the ratio of the nostril circumference of the unaffected side to that of the affected side. The closer the value is to 1, the more equal it is (Sasaki et al., 2012). The perimeter ratio of the mHP group increased from 0.98 to 1.04, while that of the NAM group decreased from 1.11 to 1.05. The perimeter ratio of the mHP group was increased, while that of the NAM group was decreased; however, there was no significant difference between the results immediately and 7 months after surgery (*p* = 0.18, *p* = 0.29, respectively; Figure 6a,b).

#### 3.2.4 Aspect a/u ratio

**Figure 7:**
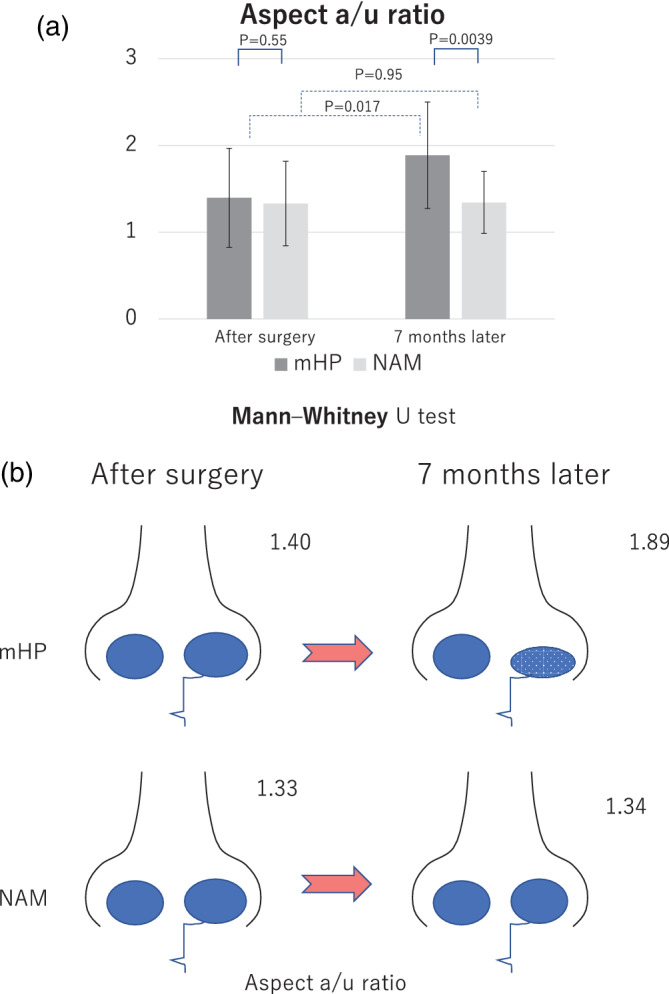
Evaluation of the surgical outcome in the modified Hotz plate (mHP) and nasoalveolar molding (NAM) groups analyzed by the aspect a/u ratio. (a) Temporal change in the surgical outcome in the two groups. mHP: mHP group (dark gray), treatment with mHP. NAM: NAM group (light gray), treatment with NAM. Results immediately and 7 months after surgery are shown. (b) Conceptual schema of the time‐series changes in the nasal morphology and relationships with the NAM and mHP groups

The aspect a/u ratio is the horizontal to vertical ratio of the affected side to the unaffected side. The closer the value is to 1, the more identical it is (Sasaki et al., 2012). The aspect a/u ratio of the NAM group decreased from 1.33 to 1.34, and there was no significant difference between the results immediately and 7 months after surgery (*p* = 0.95), whereas that of the mHP group increased from 1.40 to 1.89, and there was a significant difference (*p* = 0.017). Thus, this parameter worsened in the mHP group 7 months after surgery and not immediately after surgery, indicating asymmetric nose relapse in the mHP group after 7 months (Figure 7a,b).

#### 3.2.5 Grc‐Grn∠midline

**Figure 8:**
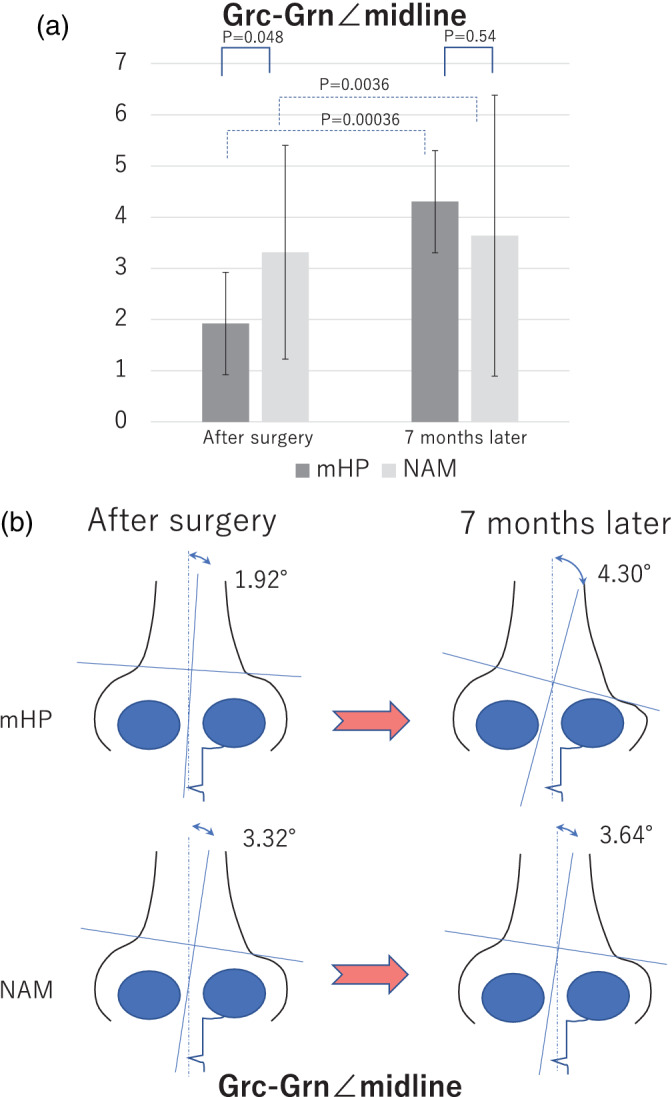
Evaluation of the surgical outcome in the modified Hotz plate (mHP) and nasoalveolar molding (NAM) groups analyzed by the Grc‐Grn∠midline. (a) Temporal change in the surgical outcome in the two groups. mHP: mHP group (dark gray), treatment with mHP. NAM: NAM group (light gray), treatment with NAM. Results immediately and 7 months after surgery are shown. (b) Conceptual schema of the time‐series changes in the nasal morphology and relationships with the NAM and mHP groups

Grc‐Grn∠midline implies the relative position of the nostril of the greater alar cartilage of the affected side to that of the unaffected side. Grc‐Grn∠midline means that the ala of the nose has collapsed, and the symmetry is lost. Values closer to 0° imply a small deviation (Adachi et al., 2013). Grc‐Grn∠midline of the NAM group increased from 3.32 to 3.64, while that of the mHP group increased from 1.92 to 4.30, 7 months after surgery. Grc‐Grn∠midline of the NAM and mHP groups increased significantly (*p* = 0.0036 and *p* = 0.00036, respectively). The greater alar cartilage in both groups relapsed; however, the magnitude of the change was remarkable in the mHP group (Figure 8a,b).

#### 3.2.6 Midline∠columellar axis

**Figure 9:**
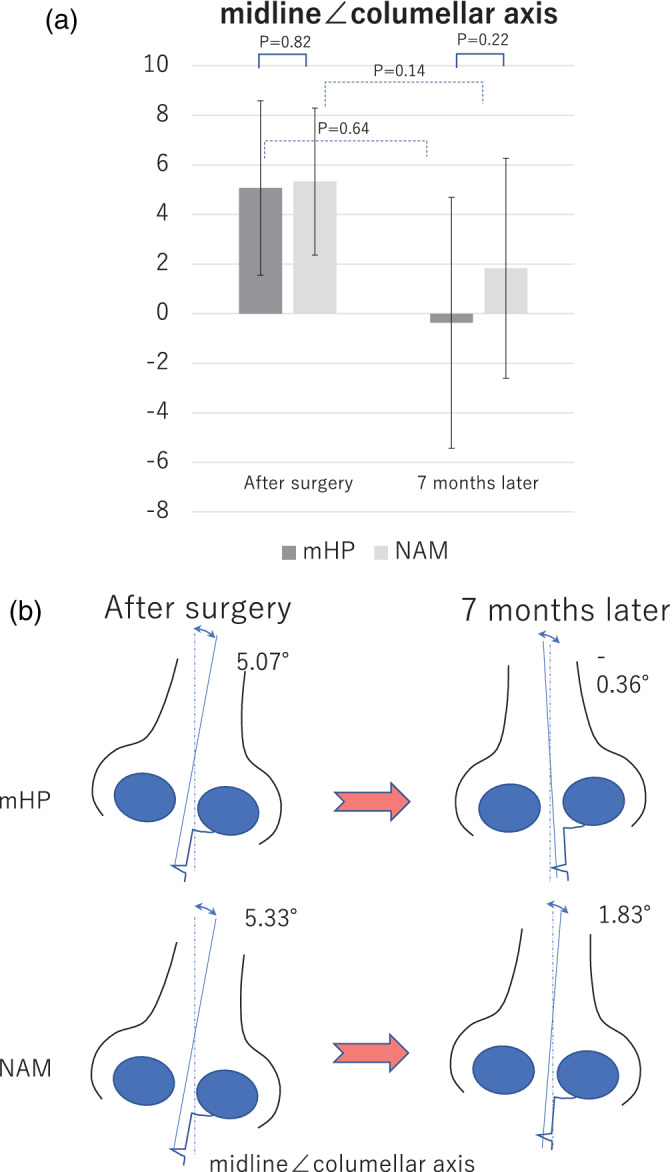
Evaluation of the surgical outcome in the modified Hotz plate (mHP) and nasoalveolar molding (NAM) groups analyzed by the midline∠columellar axis. (a) Temporal change in the surgical outcome in the two groups. mHP: mHP group (dark gray), treatment with mHP. NAM: NAM group (light gray): treatment with NAM. Results immediately and 7 months after surgery are shown. (b) Conceptual schema of the time‐series changes in the nasal morphology and relationships with the NAM and mHP groups

The midline∠columellar axis indicates the deviation of the axis between the midline and column of the nose. Values closer to 0° imply a small deviation in the direction of the column of the nose. The midline∠columellar axis of the NAM group decreased from 5.33 to 1.83, while that for the mHP group increased from 5.07 to −0.36 after surgery. There was no significant difference between the results of the NAM and mHP groups (*p* = 0.14 and *p* = 0.64, respectively; Figure 9a,b).

### 3.3 Comparison of the nostril morphology between the NAM and mHP groups

#### 3.3.1 Hausdorff distance

Although there was no significant difference in the Hausdorff distance between the NAM (*p* = 0.0049) and mHP (*p* = 0.048) groups after primary cheiloplasty (*p* = 0.77), there was a significant difference in the results (NAM, 0.0034; mHP, 0.0045) 7 months after surgery (*p* = 0.018). Thus, the effectiveness of the NAM method in shaping the nostril was significantly more sustainable and showed differences at 7 months after surgery (Figure 4a,b).

#### 3.3.2 Area ratio

There was a significant difference in the area ratio between the NAM and mHP groups after surgery (NAM, 1.17; mHP, 0.72; *p* = 0.00062), and there was no significant difference at 7 months after surgery (NAM, 0.96; mHP, 0.83; *p* = 0.79; Figure 5a,b). Thus, the area of the affected side in the mHP group was smaller than that of the normal side after surgery; however, 7 months after surgery, the relative size of the affected nostril to the normal nostril was similar.

#### 3.3.3 Perimeter ratio

There was a significant difference in the perimeter ratio between the NAM and mHP groups after surgery (NAM, 1.11; mHP, 0.98; *p* = 0.016); however, there was no significant difference 7 months after surgery (NAM, 1.05; mHP, 1.04; *p* = 0.89; Figure 6a,b). Thus, NAM treatment increased the perimeter of the affected nostril after surgery more than that of the passive orthopedic appliance; however, the result was similar 7 months after surgery.

#### 3.3.4 Aspect a/u ratio

There was no significant difference in the aspect a/u ratio between the NAM (1.33) and mHP (1.40) groups after surgery (*p* = 0.55; Figure 7a,b). At 7 months after surgery, the aspect a/u ratio was significantly lower in the NAM group (1.34) than that in the mHP group (1.89; *p* = 0.0039), reflecting that the nostrils of the patients in the mHP group relapsed and the height of the nostril decreased.

#### 3.3.5 Grc‐Grn∠midline

The mean values of the Grc‐Grn∠midline of the NAM and mHP groups were 3.32 and 1.92, respectively, without any significant difference (*p* = 0.048). At 7 months after surgery, the values in the NAM and mHP groups were 3.64 and 4.30, respectively, without any significant difference (*p* = 0.54). This means that the Grc‐Grn∠midline of patients in the NAM group was shifted more than that of patients in the mHP group; however, at 7 months after surgery, the relapse in the mHP group worsened and sustainability of the NAM group improved, with no significant difference between the results (Figure 8a,b).

#### 3.3.6 Midline∠columellar axis

The midline∠columellar axes of the NAM and mHP groups after surgery were 5.07 and 5.33, respectively, without any significant difference (*p* = 0.82). The values in the NAM and mHP groups were 1.83 and −0.36, respectively, at 7 months after surgery without any significant difference (*p* = 0.22; Figure 9a,b).

## 4 DISCUSSION

According to a computed tomography‐based study by Evteev et al. (2018), cranial growth in children is 37% of the width of an adult's palate, increasing to 67% by the age of 1 year, and reaching 77% of the adult width before the age of 5 years. In addition, the width of the nose is 51% of that of an adult at birth and increases by 43% by the age of 1 year, reaching 82% of that of an adult by the age of 5 years. Substantial skeletal changes take place during infancy, and the landmark points on bone may change significantly between the affected and control sides as the child grows. AlHayyan et al. (2018) reported a long‐term study of skeletal changes achieved using the NAM method over a period of 5 years. According to their study, the NAM method does not have a significant effect on the symmetry of the midface of children, suggesting that other important factors determine the effect of NAM. One of these factors is the shape of the nasal cartilage.

In a previous study, we compared the shape of the nostrils at 1 month after the primary cheiloplasty between a group of patients who received treatment using the NAM method and another of patients treated using a conventional appliance (Sasaki et al., 2012). This previous study showed that the NAM method improved the symmetry of the left and right nostrils. Based on the findings of this previous study, in the present study we examined how the shape of the nose changes during the approximately 7‐month period from immediately after the primary cheiloplasty and rhinoplasty to when the palatoplasty is performed.

In the current study, we evaluated the effect of the NAM method on the morphology of the nostrils and anthropometric measurements, including the Hausdorff distance, aspect ratio, perimeter diameter, and area ratio. The Hausdorff distance in the NAM group was significantly lower than that in the mHP group. On the other hand, there was no significant difference in the Hausdorff distance between the NAM and mHP groups immediately after surgery; however, 7 months after surgery, there was a significantly better symmetry in the Hausdorff distance in the NAM group. Thus, the esthetic outcome just after primary cheiloplasty was similar between the NAM and mHP groups, and the symmetry of the nostril improved with time.

The Hausdorff distance is the only factor that showed a significant difference in our analysis. We hypothesize that this finding may be due to (1) the measurement method using Hausdorff distance closely resembling human sensory evaluation and therefore having a higher power of detection than other factors (Karube et al., 2012); or (2) the Hausdorff distance being purely indicative of changes in the cartilage of the nose, since it mathematically quantifies differences in the geometric shape of the nostrils. In contrast, the Grc‐Grn∠midline and midline∠columellar axes are indicators of a specific position on the face and, as a patient develops, they move in accordance with the patients' skeletal growth and changes.

In terms of the area ratio, both groups showed improvements immediately and 7 months after surgery. However, the area ratio of the NAM group was significantly greater than that of the mHP group immediately after surgery. Neither of the groups showed any difference in the perimeter ratio immediately or 7 months after surgery; however, the perimeter ratio of the NAM group was significantly more than that of the mHP group immediately after surgery. The nostril in patients in the NAM group was made slightly larger by the surgical technique during primary cheiloplasty than that in patients in the mHP group. On the other hand, although the aspect a/u ratio did not change significantly immediately after surgery, it increased with time in the mHP group and became significantly larger after 7 months. However, as there was no change in the aspect a/u ratio in the NAM group, the value was significantly higher in the mHP group. In addition, the Grc‐Grn∠midline was significantly more displaced in the mHP group than in the NAM group immediately after surgery; however, the difference was not significant 7 months after surgery. There was also a significant increase in Grc‐Grn∠midline in both groups with time. These results indicate that the nasal axis was tilted in both groups due to collapse of the ala of the nose on the affected side, although the change in the NAM group was small due to less deformation. Hence, the inclination of the nasal axis after 7 months was similar.

In our previous paper, we showed that the Hotz plate improves upper jaw plane leveling of the nasal floor (Adachi et al., 2013). However, in the present study, there was no difference in the variation of the height of the jawbone at the base of the ala because the plate covered both jaw clefts. This was probably because the size of the ala in the collapsed nose in the NAM group was small. Supportive results show that the midline∠columellar axis, which indicates the direction of the bridge of the nose, tends to decrease in both groups as the jaw grows, without any significant difference.

Our results suggest that nasal stent treatment of the greater nasal alar cartilage from postnatal 21 to 85 days was the most critical factor for nasal esthetics. Several reports have indicated that NAM improves the outcome of nasal morphology (Barillas et al., 2009; Bennun et al., 1999; Chang et al., 2010; Clark et al., 2011; Liou et al., 2004; Pai et al., 2005). Barillas et al. reported that lower and lateral septal cartilages are more symmetric when using a cast model of the nose at the age of 9 years. This study used a surgery‐alone group as the control; however, improved nasal symmetry was observed for a long time (Barillas et al., 2009). Besides, a long‐term follow‐up study showed that NAM did not have a significant effect on the midface symmetry in children (AlHayyan et al., 2018). These reports also suggest that the critical factor of NAM is an orthodontic force to the nasal cartilage by the nasal stent in the early neonatal period because alar cartilage is correctable in the early neonatal period (Matsuo & Hirose, 1991).

This study provides a very new perspective by taking into account changes in the surgical technique, which was not considered before. The surgeon performed primary cheiloplasty to obtain more symmetrical outcomes between the affected and unaffected nasal ala in advance by using techniques such as over‐ or under‐correction of the affected region based on their own clinical experience. If the surgical technique is perfect, the surgeon will estimate the degree of deformation and operate in such a way that it reaches an adequate size when it is deformed after surgery by residual correction or overcorrection. In our study, the surgeon made the affected nostrils larger, anticipating postoperative deformities. This technique may be suitable for primary cheiloplasty with NAM treatment. Chang et al. reported that overcorrection of 20% was necessary to maintain the nostril height (Chang et al., 2010), which is similar to our results. If a precise measurement method is established, we can estimate morphological changes in the nose after growth and then modify the surgical technique to obtain better clinical outcomes. In the future, it will be necessary to establish a precise method to evaluate the differences in the surgical techniques. In addition, the precise method could be useful for determining the proper shape of the stent, direction of force application, and the duration of treatment.

There are many factors that impact final nasal morphology, and we need a proper method to assess these factors accurately. Some of the problems that may arise with final nasal morphology include cartilage shape reversion, overcorrection as a result of a surgeon's specific surgical technique, and deformation due to the skeletal growth of the infant patients. In the future, it may be necessary to use the correct evaluation method for each of these factors to evaluate relapse of the nasal wing shape.

In summary, our results showed that the symmetry of the NAM was maintained by preventing the collapse of the nasal wing, and there was no difference in the axis of the nasal pillar between the two groups. The impact of the NAM method was mainly derived from correction of the greater nasal alar cartilage modified by the nasal stent of NAM during the first 3 months after birth. There are various confounding factors and limitations found with this observational study of two CLP surgery methods, such as the small sample size, problems with the surgical technique (such as suturing and degree of overcorrection), differences in the degree of deformity in the patients, and differences in skeletal growth. The fact that the treatment method may have been modified by the patients' families or that the surgeon's skill may have improved over the duration of the study period could also have impacted on our findings. Reliable clinical studies, such as randomized controlled trials with more cases, will be necessary to confirm the efficacy of the NAM method shown in this study. If more precise measurement methods for the NAM effect are established, it will be useful for determining the stent technique of NAM and surgical design during primary cheiloplasty to assess the final morphology of the nose after growth.

## FUNDING

The authors have no sources of financial support to declare.

## CONFLICT OF INTEREST

The authors declare no conflict of interest.

## AUTHOR CONTRIBUTIONS

*Contributed to the study conception and design, data acquisition and analysis, and drafting of the manuscript*, Yukiko Aihara. *Contributed to the study conception and design, data analysis, and drafting of the manuscript*, *Contributed to the study conception and design, data analysis and interpretation, and drafting and critical revision of the manuscript*, Toru Yanagawa. *Contributed to data acquisition, analysis, interpretation, and drafting of the manuscript*, Katsuhiko Tabuchi, Hiroki Bukawa, Mitsuru Sekido. Masahiro Sasaki, Kaoru Sasaki, Yoichiro Shibuya, Koji Adachi, Shinji Togashi, Shohei Takaoka. All authors have read and agreed to the published version of the manuscript, (and any substantially modified version that involves the author's contribution to the study).

## Data Availability

The data that support the findings of this study are available from the corresponding author upon reasonable request.
